# Draft genome sequence of *Lacticaseibacillus rhamnosus* CRL 2244, a strain with strong killing effect on carbapenem-resistant *Acinetobacter baumannii*

**DOI:** 10.1128/mra.00892-23

**Published:** 2023-11-29

**Authors:** German Matias Traglia, Cecilia Rodriguez, Camila Leal, Briea Gasca, Adiba Aziz, Christina Lopez, Maria Lucia Petrelli, Nicholas T. Salzameda, Robert A. Bonomo, Raúl Raya, Maria Soledad Ramirez

**Affiliations:** 1 Unidad de Genomica y Bioinformatica, Departamento de Ciencias Biologicas, CENUR Litoral Norte, Universidad de la Republica, Salto, Uruguay; 2 Centro de Referencia para Lactobacilos (CERELA), CONICET, Tucumán, Argentina; 3 Department of Biological Science, College of Natural Sciences and Mathematics, Center for Applied Biotechnology Studies, California State University Fullerton (CSUF), Fullerton, California, USA; 4 Department of Chemistry and Biochemistry, College of Natural Science and Mathematics, CSUF, Fullerton, California, USA; 5 Research Service and GRECC, Louis Stokes Cleveland Department of Veterans Affairs Medical Center, Cleveland, Ohio, USA; 6 Departments of Medicine, Pharmacology, Molecular Biology and Microbiology, Biochemistry, Proteomics and Bioinformatics, Case Western Reserve University School of Medicine, Cleveland, Ohio, USA; 7 CWRU-Cleveland VAMC Center for Antimicrobial Resistance and Epidemiology (Case VA CARES), Cleveland, Ohio, USA; Wellesley College, Wellesley, Massachusetts, USA

**Keywords:** *Acinetobacter baumannii*, lactic acid bacteria, carbapenems

## Abstract

We report here a draft genome assembly of *Lacticaseibacillus rhamnosus* CRL 2244, recovered from wastewater in Argentina. The genome has a size of 2,898,100 bp, with G + C content of 46.73%. Comparative analysis reveals that its closest relative is *L. rhamnosus* 1.0320 (GCF_006151905.1), with an average nucleotide identity of 97.46%.

## ANNOUNCEMENT

*Lacticaseibacillus rhamnosus* is a Gram-positive bacillus with Qualified Presumed Safety status, known to inhabit a wide range of environmental niches, and has gained attention for the probiotic properties exhibited by certain strains (1).

The strain *L. rhmanosus* CRL 2244 was isolated from a wastewater sample in the year 2016 in the province of Tucumán (geographical coordinates 26°52'29.8 "S 65°12'46.4 "W), Argentina. Briefly, wastewater samples were diluted in phosphate buffered saline (PBS), filtered with a 0.45 µm pore size membrane, and placed on a sterile filter paper pad soaked with Man, Rogosa, and Sharpe (MRS) broth. The filter paper was then placed in MRS Agar (Oxoid, Hampshire, UK) containing 100 µg/mL vancomycin, cycloheximide 50 µg/mL, and sodium azide 50 µg/mL and incubated at 37°C for 24–48 h. Colonies were subsequently stained by gram staining and characterized with conventional biochemical tests for identification (2, 3).

Recently, we reported that the *L. rhamnosus* CRL 2244 strain has a strong ability to inhibit the growth and even cause the death of both susceptible and carbapenem-resistant strains of *Acinetobacter baumannii* (4).

*L. rhamnosus* CRL 2244 strain maintained at −80°C in milk yeast extract (LEL) with 20% (vol/vol) glycerol was cultured in MRS medium broth for 16 h at 37°C. *L. rhamnosus* CRL 2244 cells were lysed, and total DNA extraction was carried out using Wizard Genomic DNA Purification Kit according to manufacturer instructions (Promega, Madison, WI). Sequencing libraries were prepared using the Illumina DNA Prep kit and IDT 10 bp UDI indices according to the manufacturer’s recommendations. The whole genome of CRL 2244 was sequenced on the Illumina NextSeq 2000 (SeqCenter Inc.), generating a total of 1,347,861 paired ends with a read length of 2 × 151 bp. Raw sequencing reads were subjected to quality control and adapter trimming using fastQC v0.11.9 and Trimmomatic v0.38, respectively (5). Clean reads were then *de novo* assembled using SPAdes v3.15.4 (6), removing contigs smaller than 500 bp to ensure sequencing quality, leaving a total of 35 contigs containing 2,898,100 bp. All software was used with default settings unless otherwise specified. The maximum and minimum scaffold lengths were 349,371 and 510 bp, respectively. The scaffold N_50_ and N_90_ values were 180,622 and 52,479 bp, respectively. Overall G + C content was determined to be 46.73%.

Whole-genome average nucleotide identity (ANI) and *in-silico* DNA-DNA hybridization (DDH) were performed to correct species identification and estimate the relationship of *L. rhamnosus* CRL 2244. ANI and DDH was calculated by anib.rb (7) and GGDC (8). The ANI value of CRL 2244 compared with reference genomes of *Lacticaseibacillus* species (*n* = 28) was higher than 97% against to *L. rhamnosus* 1.0320 (GCF_006151905.1). Also, DDH comparison with reference genome of *Lacticaseibacillus* species (*n* = 28) showed a DDH higher than 70% against to *L. rhamnosus* 1.0320 (GCF_006151905.1). Genome annotation was performed by prokka software (9). A total of 2,674 CDS and 58 tRNA were identified in CRL 2244.

Antimicrobial resistance genes were identified using BLAST (*E*-value < 10^−5^) and MegaRes database (10). No genes associated with antimicrobial resistance were detected. Three homolog genes associated to virulence (*lap*, *hasC*, and *clpP*) were identified using BLAST (*E*-value < 10^−5^) and Virulence Factor Database (VFDB).

## Data Availability

This whole-genome shotgun project has been deposited at DDBJ/EMBL/GenBank under the accession no. JAVFWJ000000000.
